# Gamma-H2AX-Based Dose Estimation for Whole and Partial Body Radiation Exposure

**DOI:** 10.1371/journal.pone.0025113

**Published:** 2011-09-23

**Authors:** Simon Horn, Stephen Barnard, Kai Rothkamm

**Affiliations:** 1 Health Protection Agency Centre for Radiation, Chemical and Environmental Hazards, Chilton, Oxfordshire, United Kingdom; 2 Queen's University Belfast Centre for Cancer Research and Cell Biology, Belfast, United Kingdom; University of Medicine and Dentistry of New Jersey, United States of America

## Abstract

Most human exposures to ionising radiation are partial body exposures. However, to date only limited tools are available for rapid and accurate estimation of the dose distribution and the extent of the body spared from the exposure. These parameters are of great importance for emergency triage and clinical management of exposed individuals. Here, measurements of γ-H2AX immunofluorescence by microscopy and flow cytometry were compared as rapid biodosimetric tools for whole and partial body exposures. *Ex vivo* uniformly X-irradiated blood lymphocytes from one donor were used to generate a universal biexponential calibration function for γ-H2AX foci/intensity yields per unit dose for time points up to 96 hours post exposure. Foci – but not intensity – levels remained significantly above background for 96 hours for doses of 0.5 Gy or more. Foci-based dose estimates for *ex vivo* X-irradiated blood samples from 13 volunteers were in excellent agreement with the actual dose delivered to the targeted samples. Flow cytometric dose estimates for X-irradiated blood samples from 8 volunteers were in excellent agreement with the actual dose delivered at 1 hour post exposure but less so at 24 hours post exposure. In partial body exposures, simulated by mixing *ex vivo* irradiated and unirradiated lymphocytes, foci/intensity distributions were significantly over-dispersed compared to uniformly irradiated lymphocytes. For both methods and in all cases the estimated fraction of irradiated lymphocytes and dose to that fraction, calculated using the zero contaminated Poisson test and γ-H2AX calibration function, were in good agreement with the actual mixing ratios and doses delivered to the samples. In conclusion, γ-H2AX analysis of irradiated lymphocytes enables rapid and accurate assessment of whole body doses while dispersion analysis of foci or intensity distributions helps determine partial body doses and the irradiated fraction size in cases of partial body exposures.

## Introduction

Dose assessments based on well established cytogenetic assays and especially those utilising emerging techniques in the field of biological dosimetry are mainly suited for whole body exposures to ionising radiation [1]. However, most human radiation exposures are partial body exposures, whether during planned medical exposures or in the case of radiation accidents. Information about the extent of ‘sparing’ of normal tissues during a high dose exposure and accurate estimates of peak doses delivered to localised regions of the body are of crucial importance for the clinical management of radiation casualties [2]. To address this need, analytical methods have been developed for the long-established dicentric assay, the current ‘gold standard’ in biological dosimetry, to identify partial body exposures and calculate the irradiated fraction of the body and estimate peak doses to the irradiated fraction [3]. In contrast, many of the emerging biological dosimetry techniques, which focus on quick dose assessments to facilitate rapid triage, have not been tested as rigorously in cases of partial body exposure settings.

The phosphorylated histone H2A variant γ-H2AX and p53 binding protein 53BP1 are established immunocytochemical markers of ionising radiation-induced DNA double-strand breaks (DSBs) [4], [5] and are emerging biomarkers of radiation exposure [6], [7]. γ-H2AX and 53BP1 foci form at the sites of DSBs and can be visualised within minutes of exposure [8]. Their potential for accurately estimating radiation dose has already been reported following experimental human *ex vivo* [9]–[11], non-human primate *in vivo* [12] and diagnostic [8] or therapeutic [13] human *in vivo* exposure. These studies demonstrate excellent sensitivity down to a few milligray, the ability of the γ-H2AX assay to identify a recent partial body exposure, and persistence of foci for several days after high dose exposure.

As an alternative to scoring γ-H2AX foci by immunofluorescence microscopy, γ-H2AX can also be quantified by flow cytometry [14], [15]. Although both methods measure γ-H2AX levels using immunofluorescence assays, flow cytometry detects total fluorescence intensity in each cell whilst microscopy allows scoring of individual foci [7]. The main advantage of flow cytometry is speed; where scoring foci by eye can be laborious (although still far faster than dicentric scoring), flow cytometry can rapidly analyse thousands of cells within minutes in an unsupervised manner. Even automated foci scoring is significantly slower, as multiple fields of view, optical planes at different depths and fluorescence channels must be recorded at high spatial resolution, followed by the actual image analysis procedures for foci scoring [16]–[18]. Although the analysis speed differs between the two techniques, the sample processing and immunostaining methods are largely similar and can be completed in similar times.

For partial body exposures, estimating the fraction of the body irradiated and dose to irradiated fraction require detailed analysis of signal distributions and present a greater challenge in interpreting results. For dicentric scoring, the distribution of aberrations in the irradiated part of the lymphocyte pool plays a key role [3]. A high abundance of both cells with multiple dicentrics and cells with normal metaphases gives rise to an overdispersed distribution of aberrations which is a hallmark of partial body exposure. In contrast, whole body exposure to sparsely ionising radiation results in the random induction of aberrations in all cells and therefore follows a Poisson distribution [19]. For densely ionising radiation however, even a whole body exposure will result in a non-uniform dose distribution due to the clustered energy deposition along the particle tracks [20], making dose estimates modeled on Poisson distributions inappropriate.

Here we describe the use of γ-H2AX and/or 53BP1 immunofluorescence microscopy and γ-H2AX flow cytometry as quantitative biomarkers for whole and partial body exposure to ionising radiation.

## Results

### γ-H2AX and 53BP1 foci induction is linear and loss follows a bi-exponential decay that can be approximated with one universal calibration function

**Figure 1:**
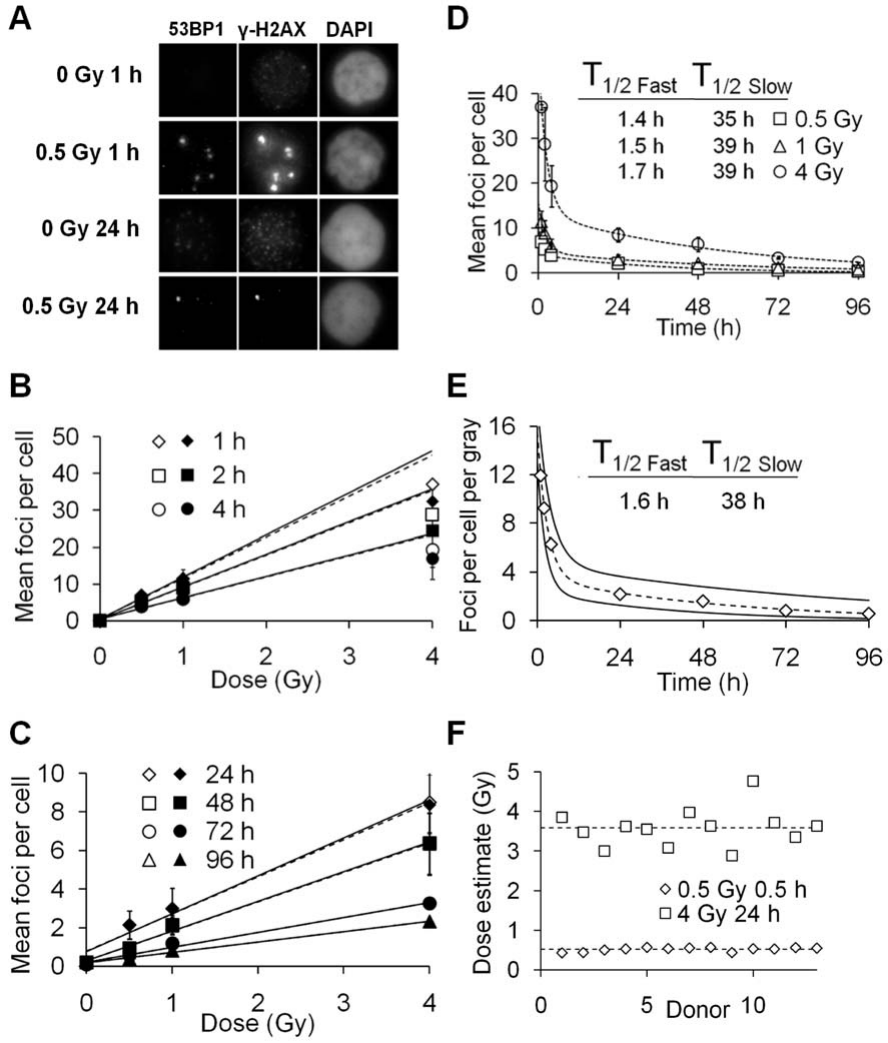
γ-H2AX (open symbols) and 53BP1 (closed symbols) foci induction and loss after uniform exposure to X-rays, detected by immunofluorescence microscopy. (A) Grey scale immunofluorescent images of 53BP1, γ-H2AX and DAPI in irradiated lymphocytes and controls. Each image is 8.4×7.6 µm in size. (B) Mean γ-H2AX and 53BP1 foci observed between 1–4 hours post exposure. Linear regressions (solid lines for γ-H2AX, dashed for 53BP1) were fitted excluding the 4 Gy data points due to underscoring of foci. (C) Mean γ-H2AX and 53BP1 foci observed between 24–96 hours post exposure. Linear regressions (solid for γ-H2AX, dashed for 53BP1) are fitted to all data points. (D) γ-H2AX foci loss over 96 hours. Dashed lines represent bi-exponential fits for γ-H2AX loss for each dose. T_1/2 Fast_ and T_1/2 Slow_ represent the individual half-lives of the two exponentials. (E) γ-H2AX foci yields per cell per gray between 1–96 hours calculated using linear regression coefficients of γ-H2AX foci counts from Figures 1B and 1C. The dashed line represents a bi-exponential fit for mean γ-H2AX loss per unit dose and solid lines the upper and lower 95% confidence limits. (F) Dose estimates in X-irradiated lymphocytes from 13 healthy donors. γ-H2AX foci were scored and the dose estimated using the calibration curve from Figure 1E. The dashed lines represent the average dose estimates based on all the donors.

In *ex vivo* irradiated human lymphocytes, γ-H2AX and 53BP1 foci colocalized (Fig. 1A) and were linearly induced by acute X-ray doses of 0.5 and 1 Gy between 1–4 hours post exposure (Fig. 1B) and 0.5, 1 and 4 Gy between 24–96 hours post exposure (Fig. 1C). Mean γ-H2AX and 53BP1 foci counts per cell were very similar for all samples except those with the highest levels of foci, i.e. 1, 2 and 4 hours post 4 Gy exposure. At 96 hours, the mean foci level for 0.5 Gy-irradiated lymphocytes was significantly increased against controls (p = 0.015, *t* test).

Similar time courses of γ-H2AX and 53BP1 foci loss over a 96 hour period after exposure to X-rays followed a bi-exponential decay with a fast and a slow component for all doses (Fig. 1D). Notably, the T_1/2 Fast_ and T_1/2 Slow_ half-lives were very similar at all three doses, suggesting foci loss and therefore DSB repair is independent of the X-ray dose received. With this information it was decided a single decay could be used to describe γ-H2AX loss over 96 hours and was accomplished as follows: Mean foci yields per gray, obtained by linear regression for each time point for γ-H2AX, were used to generate a dose-normalised biexponential γ-H2AX decay curve (Fig. 1E) which followed the function y = 11.92*exp(−0.3495*x)+3.552*exp(−0.01843*x), where y = foci yield per gray and x = time in hours. Assuming dose is proportional to foci yield, dose = foci scored/y at x hours post exposure. A Pearson's chi-square test to determine the goodness of fit of the bi-exponential decay to the linear regression coefficients for the dose response curves gave a value of p = 0.998.

To confirm the validity of the γ-H2AX foci calibration curve, human lymphocytes from a group of healthy donors were irradiated *ex vivo* with 0.5 Gy and 4 Gy and analysed at 1 and 24 hours post exposure, respectively. For foci scoring of 13 donors (Fig. 1F), the dose estimates for 0.5 Gy 1 hour samples varied between 0.43–0.56 Gy with a mean of 0.51 Gy. For 4 Gy 24 hour samples dose estimates were between 2.9–4.7 Gy with a mean of 3.6 Gy.

### γ-H2AX intensity increases linearly with dose and loss follows a bi-exponential decay that can be approximated by one universal calibration function

**Figure 2:**
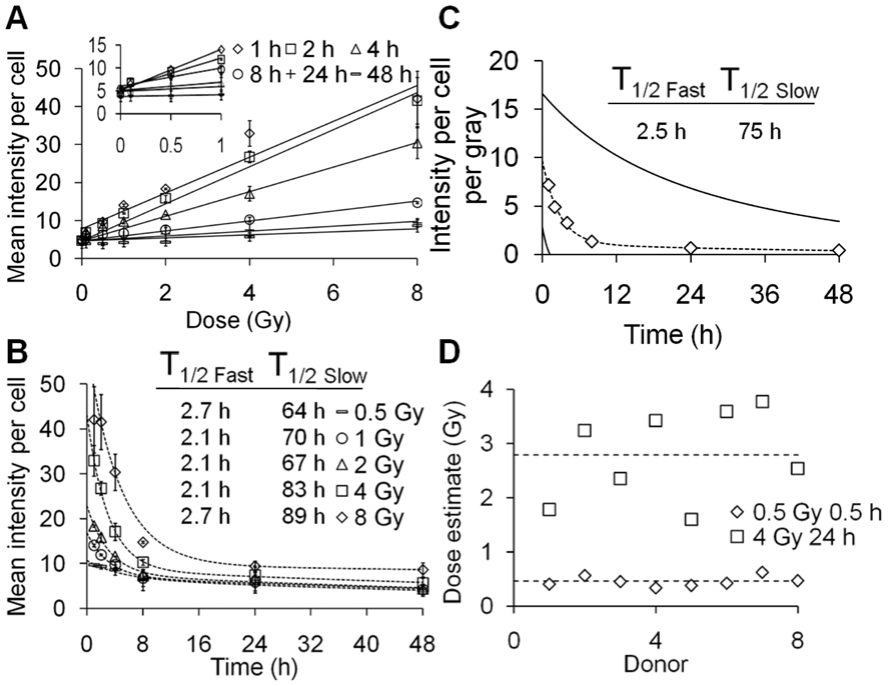
γ-H2AX intensity induction and loss after uniform exposure to X-rays, detected by flow cytometry. (A) Mean γ-H2AX intensity observed between 1–48 hours post exposure. Linear regressions (lines) were fitted to all data points. (B) γ-H2AX intensity loss over 48 hours post exposure. Dashed lines represent bi-exponential fits for γ-H2AX loss for each dose. T_1/2 fast_ and T_1/2 Slow_ represent the individual half-lives of the two exponentials. (C) γ-H2AX intensity yields per cell per gray between 1–48 hours calculated using linear regression coefficients of γ-H2AX intensity yields from Figure 2A. The dashed line represents a bi-exponential fit for mean γ-H2AX loss per unit dose and solid lines the upper and lower 95% confidence limits. (D) Dose estimates in X-irradiated lymphocytes from 8 healthy donors. γ-H2AX intensity was measured and the dose estimated using the calibration curve from Figure 2C.

γ-H2AX intensity, as measured by flow cytometry, increased linearly with dose in lymphocytes exposed *ex vivo* to 0.5–8 Gy X-rays (Fig. 2A). At 1–4 hours but not at later time points, mean intensity per cell for 0.5 Gy irradiated lymphocytes was significantly increased compared to controls (p = 0.0184, *t* test). The lowest detectable dose increased to 4 Gy at 24 hours (p = 0.0481). In contrast to γ-H2AX, 53BP1 intensity did not increase significantly after irradiation (average intensities were (15.42 vs. 14.03 vs. 14.63, for 0, 0.5 and 4 Gy, respectively, at 1 hour post exposure). As for foci loss the loss of γ-H2AX intensity over 48 hours post-exposure followed a bi-exponential decay (Fig. 2B). The T_1/2 Fast_ and T_1/2 Slow_ for intensity loss were however considerably higher than those seen for foci loss, with the T_1/2 Slow_ component increasing slightly with dose. Linear regressions for γ-H2AX intensity yields per gray for each time point were used to generate γ-H2AX calibration data (Fig. 2C) which could be described with the bi-exponential function y = 8.593*exp(−0.3709*x)+1.136*exp(−0.023235*x), where y = intensity yield per gray and x = time in hours. As dose is proportional to intensity yield, dose = intensity/y at x hours post exposure. A Pearson's chi-square test to determine the goodness of fit of the bi-exponential decay to the linear regression coefficients for the dose response curves produced a value of p = 0.999. As with foci, the γ-H2AX intensity calibration curve was used to make dose estimates in *ex vivo* irradiated human lymphocytes from a group of healthy donors. For intensity measurements in 8 donors (Fig. 2D), the dose estimates for 0.5 Gy 1 hour samples varied between 0.34–0.62 Gy with a mean of 0.46 Gy. For 4 Gy 24 hour samples dose estimates were between 1.78–3.59 Gy with a mean of 2.78 Gy.

### Overdispersion analysis detects and quantifies simulated partial body exposures in microscopic foci data sets

To determine whether microscopic foci analysis would be able to detect and characterize partial body exposures, irradiated and unirradiated lymphocytes were mixed in different ratios and the irradiated fraction size and dose to the fraction estimated using the contaminated Poisson method and γ-H2AX calibration function shown in Figure 1E.

**Figure 3:**
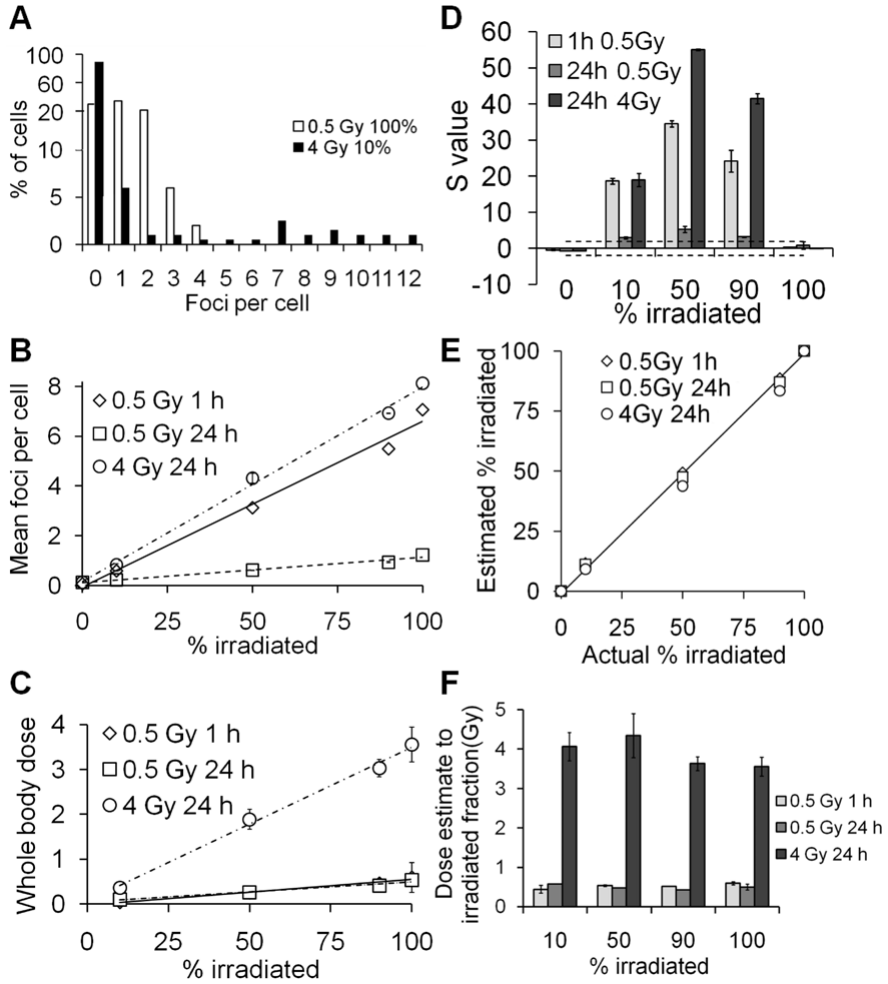
γ-H2AX foci analysis of a mixture of irradiated and unirradiated lymphocytes. (A) γ-H2AX foci distribution at 24 hours after exposure of 100% of the cells to 0.5 Gy or 10% of the cells to 4 Gy; mean foci per cell were 0.975 and 0.905, respectively. Note the logarithmic y-axis scale. (B) Mean γ-H2AX foci per cell as a function of the irradiated fraction. Linear regressions were fitted to all data points. (C) Equivalent whole body dose estimates based on mean foci counts from Figure 3B and foci calibration curve presented in Figure 1E. (D) Poisson S values calculated for γ-H2AX foci distributions in partially irradiated lymphocytes. Dashed lines mark the significance thresholds of −1.96 and 1.96. (E) Estimated versus actual irradiated fraction for the different simulated partial body exposures. Estimated fraction was calculated using the contaminated Poisson method. The line indicates a one-to-one correlation. Values have been corrected for background. (F) Dose estimates to the irradiated fraction.

Whilst different doses given to different fractions of the lymphocyte pool can result in comparable mean foci numbers, the distributions of these foci within the cell samples vary considerably (Fig. 3A). Increasing the irradiated fraction size linearly increased the mean number of foci per cell (Fig. 3B). At 1 hour, a dose of 0.5 Gy to 10% of the lymphocyte population significantly increased mean foci per cell against controls (p = 0.0129, *t* test), whereas at 24 hours, a dose of 0.5 Gy to 50% of the lymphocyte population was required to significantly increase the mean foci per cell against controls (p = 0.0153, *t* test). Using the calibration curve presented in Figure 1E, whole body dose estimates were made based on the mean foci per cell scored (Figure 3C). A dose of 4 Gy to 50% of the lymphocyte population at 24 hours post exposure gave a mean value of 4.32 foci per cell. Using: y = 11.92*exp(−0.3495*24)+3.552*exp(−0.01843*24) = 2.2 foci per gray at 24 h, the calibration function delivered a whole body dose estimate of 4.32/2.28 Gy = 1.96 Gy.

The Poisson *s* values for every partially irradiated sample were greater than 1.96, whereas wholly irradiated samples had *s* values between −1.96 and 1.96 (Fig. 3D). As partial body exposures were therefore detectable, the next step in dose estimation was to calculate the irradiated fraction size. Using the contaminated Poisson method the irradiated fraction of lymphocytes was estimated and compared to the actual irradiated fraction (Fig. 3E). This method accurately predicted the irradiated fraction for 0.5 Gy 1 hour, 0.5 Gy 24 hours and 4 Gy 24 hours (p = 0.96, p = 0.988 and p = 0.86 respectively, Pearson's chi-square goodness of fit test). For determining the dose to the irradiated fraction, mean foci per cell in the irradiated fraction was calculated by: fN = N(1/fP), where fN = mean foci per cell in the irradiated fraction, N = mean foci per cell and fP the irradiated fraction size calculated by contaminated Poisson.

For 4 Gy/24 hours/50% irradiated, the calculations for two independent experiments were 4.1(1/0.45) = 9.02 and 4.55(1/0.42) = 10.82, giving a mean value of 9.92 foci per cell. The dose was then calculated using the calibration function in Figure 1E: Dose to irradiated fraction = 9.92/2.28 Gy = 4.3 Gy. Using this method, satisfactory dose estimations were made for all doses and time points (Fig. 3F).

In addition to the contaminated Poisson algorithm, the Qdr method, first proposed by Sasaki and Miyata [21] and more recently suggested for γH2AX foci analysis by Redon et al. [22], was applied to the data in Figure 3. However, these methods for estimating irradiated fraction size and the mean foci per cell in this fraction (termed *F*γH2AX and *Q*γH2AX respectively [22]) were were generally found to be less accurate than the contaminated Poisson approach. A Pearson's chi-square goodness of fit test gave p values for *F*γH2AX of p = 0.75 (0.5 Gy 1 h), p = 1.1×10^−7^ (0.5 Gy 24) and p = 0.12 (4 Gy 24 h) and for *Q*γH2AX p = 0.99, p = 0.8 and p = 0.69 for the same irradiation conditions.

### Overdispersion analysis detects and quantifies simulated partial body exposures in flow cytometric intensity sets

**Figure 4:**
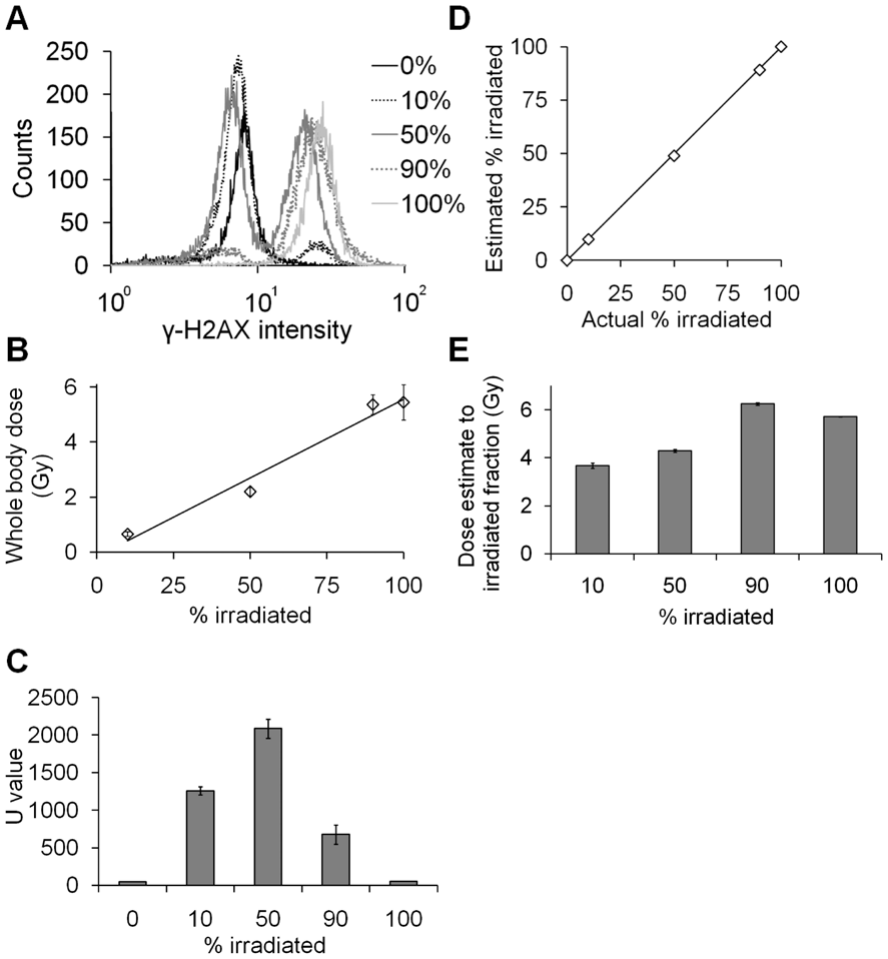
Flow cytometric γ-H2AX intensity analysis of a mixture of 4 Gy-irradiated and unirradiated lymphocytes. (A) γ-H2AX intensity histograms for different irradiated fractions at 1 hour post exposure. (B) Equivalent whole body dose estimates based on mean intensity measurements from Figure 4A and the intensity calibration function presented in Figure 2C. (C) Poisson U values calculated for γ-H2AX intensity distributions in partially irradiated lymphocytes. (D) Estimated versus actual irradiated fraction for the different partial exposures. Estimated fraction was calculated using the contaminated Poisson method. The line indicates a one-to-one correlation. (E) Dose estimates to the irradiated fraction.

Flow cytometric γ-H2AX intensity measurements in *ex vivo* partially irradiated human lymphocytes and subsequent calculations were performed in a similar way as those for foci scoring. For all partially 4 Gy-irradiated lymphocyte samples, two distinct cell populations representing unirradiated and irradiated cells were distinguishable in γ-H2AX intensity histograms (Fig. 4A). At 1 hour post exposure, a dose of 4 Gy to 10% of the lymphocyte population significantly increased the mean intensity per cell above background (p = 0.0063, *t* test). Using the calibration curve presented in Figure 2C, whole body doses were estimated based on the mean intensity per cell measured (Fig. 4B) as described previously for foci data.

Poisson *u* values were at least 13 fold greater for partially 4 Gy-irradiated samples than for uniformly irradiated samples (Fig. 4C), indicating a much greater level of over-dispersion in partially irradiated samples. The contaminated Poisson method was again used to estimate the irradiated fraction of lymphocytes which is compared to the actual irradiated fraction in Figure 4D. This method accurately predicted the irradiated fraction size (p = 0.999, Pearson's chi-square goodness of fit test). The dose to the irradiated fraction was calculated as described previously for foci data. Using this method dose estimations were made for all points (Fig. 4E).

## Discussion

Through immunofluorescence analysis, γ-H2AX and 53BP1 have been shown to be accurate and easily quantifiable biomarkers of ionising radiation exposure. Fluorescence microscopy certainly possesses the required sensitivity [8], with sub-acute doses being detectable days after exposure (Fig. 1). Flow cytometry on the other hand, whilst capable of detecting increased γ-H2AX levels shortly after sub-acute irradiation, does not maintain the required level of sensitivity beyond the first hours (Fig. 2) and appears to show wider inter-individual variation [15], [23]. Consequently, there is only very limited scope for the application of flow cytometry as a γ-H2AX biodosimetry tool (outside of planned exposure scenarios like radiotherapy). The problem appears to be intrinsic: while the eye (or image analysis software) can pick out a focus clearly from the non-specific background staining of a cell nucleus, flow cytometry measures both background and the focus intensity, resulting in increased ‘noise’ [7]. It has been reported that staining for γ-H2AX in unfixed cells increases sensitivity [23], but we have not yet been able to successfully repeat this protocol. While sensitivity is an issue, flow cytometry is a rapid and highly automated technique capable of scoring tens of thousands of cells within minutes. These qualities may be useful in the detection and quantification of other radiation biomarkers [9], [24].

The linear induction of γ-H2AX foci levels or intensity and 53BP1 foci with dose observed here and elsewhere suggests that foci and intensity yields per gray are constant; increasing the X-ray dose linearly increases the number of electron tracks and ionisations that produce DSBs in the cell. At 4 Gy 1–4 hours post exposure foci counts for both γ-H2AX and 53BP1 do not conform to linearity. This ‘underscoring’ of foci at high doses early after exposure is likely caused by the close proximity or even overlap of adjacent foci which makes it impossible to distinguish each individual focus using microscopy. For 53BP1, which relocalises to DSBs, intensity does not increase after radiation exposure (see Results); foci numbers do increase but deviate from linearity at doses above ∼2 Gy at early time points. This suggests that more DSBs result in fewer proteins accumulating at each break site, causing the dimming of individual foci as fewer antibodies bind at each focus in situations of high DNA damage levels. This is the main reason why γ-H2AX and not 53BP1 was chosen to generate dose calibration curves and subsequent dose estimates in this study. However, it is still advantageous to co-stain with both to reduce the possibility of scoring fluorescent antibody aggregates (visible in only one fluorescent channel but not the other) as foci.

The kinetics of γ-H2AX foci and intensity and 53BP1 foci loss over 96 hours were similar for all investigated doses. This suggests that DSBs are repaired independently of each other, highlighted by the similar half-lives observed between 1 and 4 Gy for γ-H2AX loss. Bi-exponential loss of γ-H2AX and 53BP1 confirms the concept that DSBs are repaired by a ‘fast’ and a ‘slow’ pathway [25], the majority being repaired by the fast pathway within 4 hours of their formation (Fig. 1C).

**Figure 5:**
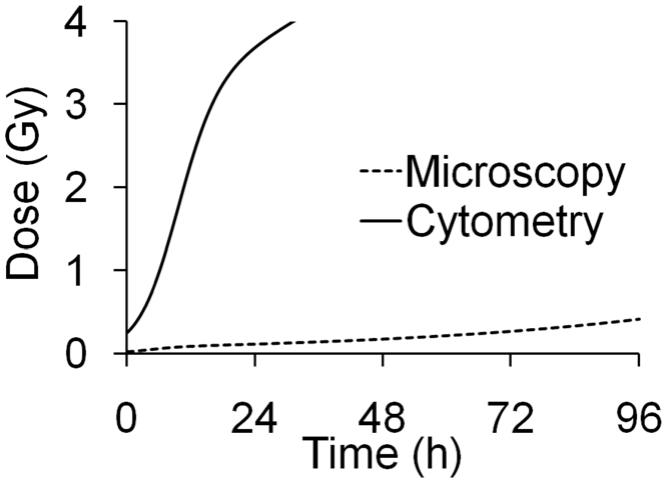
Minimum detectable dose for microscopic vs. flow cytometric γ-H2AX biodosimetry over 96 hours post exposure.

γ-H2AX dose calibration curves based on a single donor not only accurately estimated doses for that donor, but also for many others, suggesting low intra- and inter-donor variations in γ-H2AX induction and loss, one of the main strengths of cytogenetic biodosimetry [3]. For microscopy and cytometry, 0.5 and 4 Gy (non-lethal and potentially lethal doses respectively) could be distinguished, and in no case would an incorrect classification have been made. The γ-H2AX calibration curve assumes a background level of zero foci per cell. All the donors included in this study had spontaneous foci levels of <0.2 per cell; accordingly at 24 hours post exposure a level of 0.2 foci per cell would give a dose estimate of ∼100 mGy. It should be noted that background levels of spontaneous foci observed here and in published *in/ex vivo* studies using peripheral blood lymphocytes [8], [11], [12] or other primary human quiescent cells [26] are lower than those reported for established cell lines, especially those derived from tumours (e.g. [27]). This may be because peripheral blood lymphocytes are always in the G_0_ phase of the cell cycle, so foci formation due to replication stress, seen in rapidly dividing cell lines, will not contribute to background levels. A minimum detectable dose for whole body exposures for both microscopy and cytometry was generated assuming a background mean foci/intensity per cell was set at ≥0.2/6 respectively. The significant divide in the minimum detectable dose between γ-H2AX microscopy and flow cytometry is highlighted in Figure 5.

γ-H2AX and 53BP1 foci are considered to be accurate markers for DNA DSBs and the loss of a foci represents the repair of DSB. The kinetics of foci loss over time presented here, however, deviate somewhat from the reported kinetics of DSB repair in cell lines analysed by pulsed-field gel electrophoresis (PFGE) [25]. The slower repair times seen with foci scoring may result from some DSBs (or heat labile sites that were recorded as DSBs in previous studies [28]) being repaired within minutes of their formation such that no visible foci can form.

In the frequent cases of partial body exposures, whole body dose estimates will not only underestimate the peak dose delivered to part of the body but will potentially result in the incorrect treatment of a patient. Doses of 4 Gy or more will severely damage the bone marrow and may thus necessitate bone marrow transplantation; however if a small fraction of the bone marrow has been spared from the exposure then this risky procedure may not be required with treatment instead focusing on stimulating the surviving haemopoietic cells to replenish those lost [29]. Highly localised exposures to large doses, on the other hand, may result in severe tissue damage which could potentially be prevented or reduced by early clinical intervention [2]. Dispersion analysis of γ-H2AX foci and intensity in partially irradiated lymphocytes not only detected partial body exposure but also provided an estimate of the size of the irradiated fraction. Even in cases where mean foci per cell were only slightly raised over 0.2, for example for 0.5 Gy delivered to 10% of the lymphocytes, analysed 24 hours post exposure, the Poisson *s* value increased above 1.96, indicating an over-dispersion of foci (−0.75 vs 3.23). Separate analysis of only the irradiated cells resulted in a significant increase in foci numbers versus background and enabled estimation of the partial body dose (Fig. 3). Dose estimates for the irradiated fraction, based only on cells containing foci, were largely accurate and more so than for flow cytometry measurements. The good correlation of estimated irradiated fractions and doses with actual values also suggests that few, if any, foci were induced through bystander-type effects in unirradiated cells under *ex vivo* conditions [30].

For protracted exposures, dose estimates made within a few hours after the end of the exposure would be highly inaccurate due to the rapid rate at which the majority of γ-H2AX foci are lost. Dose estimates made over 8 hours post exposure however would have greater accuracy as only γ-H2AX foci lost through slow repair kinetics are left at this time where the difference between a protracted and acute exposure would be less evident. For protracted exposures lasting over 24 hours γ-H2AX foci scoring would not be an appropriate method for dose estimation. As different types of ionising radiation may have different foci yields and repair kinetics (e.g. [31]), individual γ-H2AX calibration curves have to be established, similar to the situation for the dicentric assay for biological dosimetry [3]. More work is needed in this area to fully establish the impact of radiation quality on γ-H2AX foci induction and their repair.

For rapid biodosimetry in a triage scenario following unplanned radiation exposure, microscopic scoring of γ-H2AX and 53BP1 foci offers great potential. Its satisfactory sensitivity up to several days post exposure, ability to determine critical partial body exposure and low inter and intra-variation in donors clearly outweigh any benefits that flow cytometry offers in terms of automation and throughput. γ-H2AX calibration data from a much larger cohort of subjects would allow for 95% confidence limits to be refined to improve current biological and statistical uncertainties. Induction of γ-H2AX foci in X-irradiated lymphocytes is very similar in both *in vivo* and *ex vivo* settings [8], [13], while persistent γ-H2AX foci have been observed up to 9 days post exposure to X-rays in non-human primate models [12]. It also remains to be seen to which extent variations in γ-H2AX levels may be indicative of individual radiosensitivity. Whilst such a correlation has been reported for cases with severe genetic DNA repair deficiencies such as Ataxia Telangiectasia [32] or Ligase IV Syndrome [6] as well as in some non-syndromic radiotherapy patients [33], others have reported a lack of correlation [34], [35]. Further studies of γ-H2AX and 53BP1 in clinical and non-clinical settings may further improve current uncertainties in dose estimation and clarify their association with individual clinical outcome.

## Materials and Methods

### Blood collection and irradiation

After obtaining ethical approval from the Berkshire research ethics committee (Ref 09/H0505/87) and informed consent from donors, peripheral blood from 21 healthy donors (no previous medical radiation exposures, aged 24–65, 11 males and 10 females) was collected into EDTA vacutainer tubes. Blood from the same donor were used for all experiments except Figures 1F and 2D. Blood was mixed with an equal volume of PBS and lymphocytes were isolated using Histopaque-1077 solution (Sigma-Aldrich) and resuspended in minimum essential medium (supplemented with 10% foetal calf serum, 1% L-glutamine and 1% penicillin/streptomycin) at 37°C. Cells were then irradiated with different doses of Cu/Al-filtered 250 kVp X-rays at 1.7 Gy/min and mixed with unirradiated lymphocytes to contain 0, 10, 50, 90 and 100% irradiated cells. Cells were incubated at 37°C, 5% CO_2_ in a humidified atmosphere for up to 4 days.

### Fixation, staining and scoring of γ-H2AX and 53BP1 foci

Cells were spotted onto glass Superfrost Plus slides (VWR international) at a concentration of ∼1×10^6^ cells/ml, fixed using 2% formaldehyde/PBS for 10 minutes, washed in PBS, permeabilised using 0.5% (v/v) Triton-X/PBS for 10 minutes then washed in PBS. Blocking was achieved using 1% (w/v) bovine serum albumin (BSA) in PBS for 30 minutes. Cells were then incubated with a combination of 1∶500 mouse monoclonal anti-γ-H2AX antibody (Abcam) and 1∶400 rabbit polyclonal anti-53BP1 antibody (Bethyl Laboratories) in 1% BSA/PBS for 1 hour at room temperature. Cells were then washed in 1% BSA/PBS, incubated in 1∶200 anti-mouse AlexaFluor 488 conjugated antibody (Invitrogen), 1∶200 anti-rabbit tetramethyl rodamine isoiocyanate (TRITC) conjugated antibody (Jackson ImmunoResearch) and 200 ng/ml 4′,6-diamidino-2-phenylindole (DAPI) in 1% BSA/PBS for 1 hour at room temperature, washed in PBS, dried, mounted with a cover slip using Vectashield (Vector Laboratories) and sealed using nail polish. γ-H2AX and 53BP1 foci were scored by eye with a Nikon Eclipse TE200 epifluorescence microscope using a ×100 objective with 1.3NA. Fifty cells were scored for uniformly irradiated samples and two hundred for partially irradiated samples.

### Fixation, staining and measurements of γ-H2AX intensity

Cells analysed using flow cytometry for γ-H2AX were prepared using the same protocol as those for microscopy except that all steps were performed in centrifuge tubes in suspension, not on slides. Propidium iodide was used instead of DAPI for DNA staining, and only γ-H2AX staining was used. Fluorescence intensities were quantified using a FACSCalibur flow cytometer (Becton Dickinson). Individual G_0_ lymphocytes with intact nuclei were selected and analysed based on their DNA signal (propidium iodide intensity), forward scatter and side scatter with a minimum of 5000 cells measured for each experiment. As 53BP1 relocalises to DSBs after exposure to ionising radiation (total protein levels do not increase), detection using flow cytometry is not applicable.

### Calculation of over-dispersion and irradiated fraction size

To test for over-dispersion and deviation from a Poisson distribution in γ-H2AX/53BP1 foci numbers in partially irradiated lymphocytes, the zero contaminated Poisson test or *s* test was used. The *s* test takes into account the large number of cells with zero foci that will appear in partially irradiated lymphocytes [36]. For γ-H2AX intensity measurements, the *u* test was used, which measures the deviation of the signal distribution in the sample from a Poisson distribution [37]. The *u* test was chosen instead of the *s* test as even unirradiated cells have a small degree of fluorescence. For both the *s* and *u* test, values below −1.96 indicate under-dispersion, between −1.96 and 1.96 a Poisson distribution and over 1.96 an over-dispersion. Dolphin's contaminated Poisson model was then used to calculate an estimate of the fraction of lymphocytes irradiated, and these estimates were compared with the actual irradiated fraction size [38].

### Statistical Analysis

All error bars represent the standard deviation of two independent experiments. For the *t*-test, foci and intensity levels were compared against background values taken at the same time from the same donor. Pearson's chi-square test was used to determine the goodness of fit.
